# Reappraising heme oxygenase-1 as a ferroptosis modulator in atherosclerosis: a mechanism-focused review

**DOI:** 10.3389/fimmu.2026.1737751

**Published:** 2026-02-04

**Authors:** Jia Xu, Che Chen, Han-Ying Yuan, You-Yu Zhang, Chang-Rong Wang, Xuan Xi, Heng Pan, De-Hong Li, Yan Lu

**Affiliations:** 1 School of Public Health, Gansu University of Chinese Medicine, Lanzhou, Gansu, China; 2 School of Clinical Medicine, Fudan University, Shanghai, China; 3 Department of Laboratory and Blood Transfusion, No.944 Hospital of Joint Logistics Support Force, Jiuquan, Gansu, China; 4 Department of Blood Transfusion, Gansu Provincial Hospital, Lanzhou, Gansu, China; 5 Department of Clinical Laboratory, Gansu Provincial Hospital, Lanzhou, Gansu, China

**Keywords:** atherosclerosis, ferroptosis, heme oxygenase-1, oxidative stress, ROS

## Abstract

Atherosclerosis is the primary pathological basis of cardiovascular diseases, with macrophage dysfunction, lipid accumulation, and oxidative stress driving plaque formation and progression. Ferroptosis, an iron-dependent form of regulated cell death characterized by lipid peroxidation, has recently emerged as a pivotal mechanism influencing atherosclerosis. Heme oxygenase-1 (HO-1), a key regulator of heme catabolism and iron homeostasis, exerts dual roles in this process: moderate HO-1 activity confers cytoprotection through antioxidant effects, whereas excessive HO-1 expression promotes intracellular iron accumulation, oxidative stress, and ferroptotic cell death. In macrophages, HO-1 mediates both classical ferroptosis pathways via glutathione peroxidase 4 (GPX4) regulation and non-classical, erythrophagocytosis-induced ferroptosis, contributing to plaque instability. This review systematically examines the molecular mechanisms underlying HO-1-induced ferroptosis in atherosclerosis, emphasizing its interplay with iron metabolism, oxidative stress, and macrophage function. Understanding the context-dependent effects of HO-1 provides novel insights into the regulation of vascular cell fate and plaque stability, highlighting potential therapeutic targets for the prevention and treatment of atherosclerotic cardiovascular diseases.

## 1 Introduction

Atherosclerosis is a chronic vascular disease characterized by lipid deposition and fibrous tissue proliferation in the arterial wall, and it serves as the primary pathological foundation for cardiovascular diseases (CVDs) (1). Epidemiological data indicate that most cardiovascular-related deaths are attributable to atherosclerosis-associated conditions, with coronary artery disease (49.2%) and stroke (approximately one-quarter) being the most common outcomes (2). The pathological lesions in atherosclerosis typically begin in the intima of the arteries, where lipid accumulation and inflammatory responses play pivotal roles. Under the influence of risk factors such as hyperlipidemia, hypertension, smoking, and elevated low-density lipoprotein (LDL) levels, endothelial cells undergo varying degrees of damage (3). This leads to the recruitment of vascular smooth muscle cells and circulating monocytes, which engulf oxidized low-density lipoprotein (ox-LDL), forming lipid-laden foam cells and promoting plaque formation (3, 4). Macrophages, as the predominant immune cells in plaques, play a critical role in modulating pro-inflammatory and anti-inflammatory mechanisms at various stages of plaque development (5).

Classic cell death pathways, including necrosis, apoptosis, and autophagy, contribute significantly to the pathogenesis of atherosclerosis by expanding the necrotic core and releasing pro-inflammatory cytokines (6). However, these traditional mechanisms do not fully explain the iron metabolism abnormalities, lipid peroxidation, and lipid core expansion observed in atherosclerosis. Ferroptosis, an iron-dependent regulatory form of cell death, has gained increasing attention in recent years (7). Ferroptosis not only alters the function of endothelial and smooth muscle cells but also, by modulating macrophage activity, promotes foam cell formation, further destabilizing plaques and profoundly influencing the progression of atherosclerosis (6, 7).

Among the regulatory factors of ferroptosis, heme oxygenase-1 (HO-1) has recently emerged as a key player, garnering significant attention (8). Known for its role in antioxidant stress, HO-1 catalyzes the degradation of heme to release free iron, carbon monoxide, and biliverdin, helping to maintain iron homeostasis and exert antioxidative effects (9). In atherosclerosis-related cells, HO-1 plays a key role in inhibiting disease progression through its antioxidative effects, improving endothelial cell function, modulating macrophage polarization, and inhibiting the proliferation and migration of vascular smooth muscle cells (10, 11). However, growing clinical and pathological evidence suggests that excessive upregulation of HO-1 can result in iron accumulation, promote oxidative stress, and trigger ferroptosis, destabilizing plaques (12, 13). Moreover, HO-1 expression is positively correlated with the severity of atherosclerosis (12). Under conditions of high iron load and chronic inflammation—particularly in late-stage triggering receptor expressed on myeloid cells 2 (TREM2^low) foam macrophages, which are more prone to ferroptosis—overexpression of HO-1 can lead to mitochondrial dysfunction, increasing cellular vulnerability to oxidative damage (cell viability reduced by approximately 30%) (14). Recent studies have shown that in the later stages of atherosclerosis, HO-1 participates in a non-classical ferroptotic pathway, contributing to the expansion of the necrotic core and raising the risk of plaque rupture (15, 16).

This review will explore the dual role of HO-1 in ferroptosis within atherosclerosis, with an emphasis on its involvement in iron metabolism, oxidative stress, and plaque stability. These insights provide a deeper understanding of atherosclerosis pathophysiology and highlight potential targets for novel therapeutic strategies.

## 2 Ferroptosis: molecular features and its relevance in atherosclerosis

Ferroptosis, a form of iron-dependent programmed cell death identified in 2012, is characterized by the accumulation of lipid hydroperoxides, ultimately leading to cellular damage and death (7, 17). This process arises from an imbalance between oxidative stress and cellular antioxidant defenses, particularly through the inhibition of glutathione peroxidase 4 (GPX4). As a critical enzyme involved in the detoxification of lipid hydroperoxides, the inhibition of GPX4 leads to the accumulation of lipid peroxides, which damage cell membranes and trigger ferroptotic cell death (6, 18). Morphologically, ferroptosis is marked by mitochondrial shrinkage, increased membrane density, and the loss or reduction of mitochondrial cristae (7).

**Figure 1:**
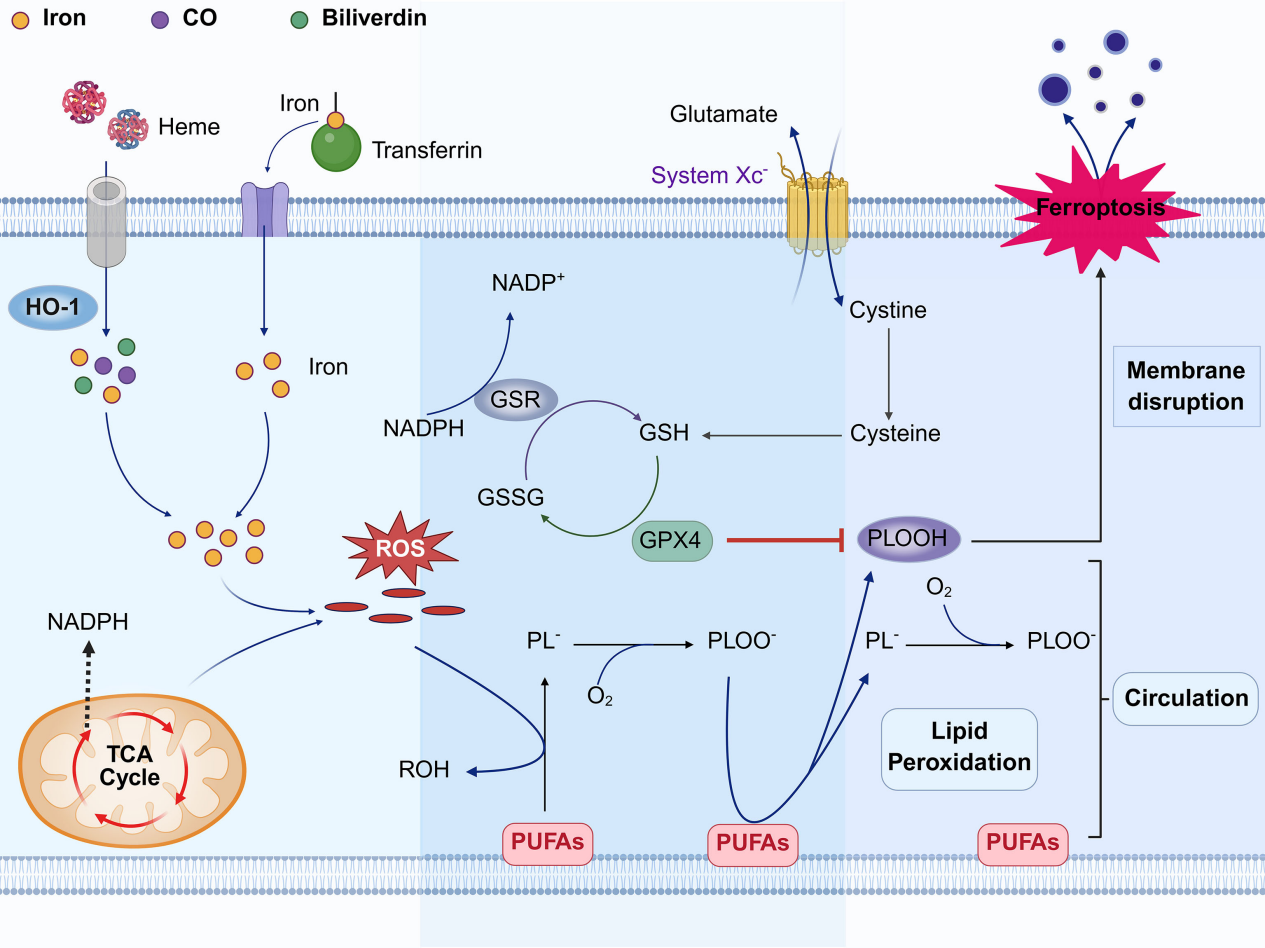
Diagram of the mechanism of ferroptosis in atherosclerosis. Iron overload during iron-induced cell death is not solely due to iron influx into the cell but also involves heme breakdown by HO-1. Excess intracellular iron leads to the generation of ROS through the Fenton reaction. These ROS abstract hydrogen atoms from the methylene carbon of polyunsaturated fatty acids (PUFAs) to form lipid radicals (PL^-^), which rapidly react with molecular oxygen to yield lipid peroxyl radicals (PLOO^-^). Subsequently, PLOO^-^ can abstract hydrogen atoms from other PUFAs, generating lipid hydroperoxides (PLOOH) and new PL^-^. The cytotoxicity of PLOOH contributes to cell damage by accumulating on the cell membrane during the lipid peroxidation chain reaction of PUFAs. This accumulation disrupts the cell membrane structure, reduces fluidity, increases permeability, and ultimately leads to membrane rupture, culminating in cell death, known as iron-induced cell death. In normal individuals, the ferroptosis process is counteracted by GPX4. Cystine is internalized into cells via cystine-glutamate antiporters (System Xc^-^) located on the cell membrane, where it is enzymatically converted to cysteine. Cysteine, a crucial precursor for glutathione biosynthesis, is utilized to generate glutathione at the expense of nicotinamide adenine dinucleotide phosphate (NADPH). Glutathione exists in two redox states: reduced glutathione (RGSH) and oxidized glutathione (GSSH). GPX4, functioning as an antioxidant enzyme, catalyzes the oxidation of GSH to GSSH, concurrently converting toxic PLOOH to harmless phospholipid alcohol. Simultaneously, glutathione reductase (GSR) regenerates GSH from GSSH using NADPH, thereby maintaining a redox equilibrium between GSSH and GSH *in vivo*. This equilibrium effectively hinders ferroptosis and ensures the normal cellular functions and homeostasis of the organism. (Created in BioRender. Li, D. (2025) https://BioRender.com/bhtcm6k).

The classical ferroptosis pathway is regulated by the Cystine/GSH/GPX4 axis. Disruption of this pathway induces oxidative stress and lipid peroxide accumulation, ultimately resulting in cell death (19, 20) (**Figure 1**). In contrast, non-classical ferroptosis operates independently of this axis. Although iron accumulation still plays a critical role in non-classical ferroptosis, the mechanisms of oxidative stress are more complex and involve a wider array of regulatory molecules and signaling pathways. For instance, iron transporters, lipid peroxidases, and inflammatory cytokines are key regulatory molecules in this pathway, collectively contributing to lipid peroxidation and ferroptosis (21). While significant progress has been made in understanding non-classical ferroptosis, it remains an emerging area of research, and a consensus definition for this process has not yet been established.

**Figure 2:**
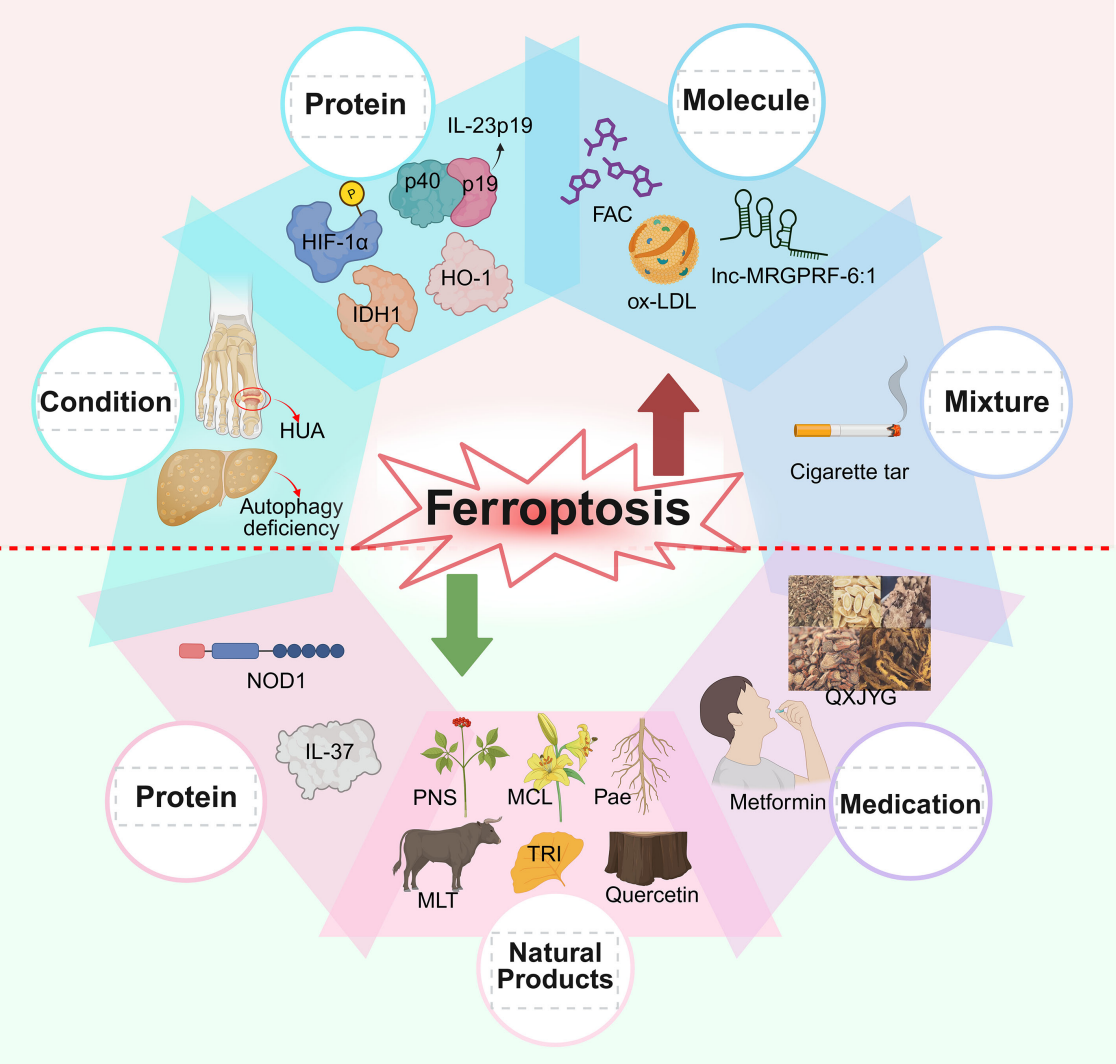
Effect of various substances on ferroptosis. Ferroptosis-promoting factors include cigarette tar, hypoxia-inducible factor 1α (HIF-1α), high levels of uric acid (HUA), *lnc-MRGPRF-6:1*, isocitrate dehydrogenase 1 (IDH1), interleukin-23p19 (IL-23p19), high expression of HO-1, oxidized low-density lipoprotein (ox-LDL), autophagy deficiency, and ferric ammonium citrate (FAC). The inhibitory factors of ferroptosis include micheliolide (MCL), interleukin-37 (IL-37), tricetin (TRI), Qing-Xin-Jie-Yu Granule (QXJYG), melatonin (MLT), quercetin, paeonol (Pae), Panax notoginseng saponins (PNS), nucleotide-binding oligomerization domain 1 (NOD1), and metformin. (Created in BioRender. Li, D. (2025) https://BioRender.com/rw147pt).

In macrophages, ferroptosis is regulated by several interrelated pathways, including the Keap1-Nrf2, system Xc^-^/GSH/GPX4, FSP1-CoQ10-NAD(P)H, and GTP-GCH1-BH4 pathways (22). In addition to these pathways, various factors influence ferroptosis in different cell types, affecting the process in both physiological and pathological contexts. Stressors such as elevated uric acid levels and autophagy deficiencies have been shown to promote ferroptosis by inhibiting the expression of protective genes regulated by the key transcription factor nuclear factor erythroid 2–related factor 2 (Nrf2) (23, 24). In contrast, compounds like micheliolide, interleukin-37, melatonin, and certain Chinese herbal medicines exert protective effects by activating Nrf2, thereby enhancing the expression of GPX4 and reduced glutathione (GSH) and inhibiting ferroptosis (25–33). Conversely, agents such as ox-LDL, ferric ammonium citrate, and *lnc-MRGPRF-6:1* interfere with GPX4 function or modulate ferroptosis-related signaling pathways, facilitating lipid peroxidation and ferroptotic cell death (34–41) (**Figure 2**).

In-depth exploration of these molecular mechanisms provides critical insights into macrophage ferroptosis and identifies potential therapeutic targets for atherosclerosis.

## 3 HO-1: from heme degradation to ferroptosis modulation

HO-1, encoded by *HMOX1*, is a heme-metabolizing enzyme predominantly expressed in the spleen and reticuloendothelial system (42). Under inflammatory, infectious, or oxidative stress stimuli, HO-1 expression is significantly upregulated, leading to increased synthesis and release (43). In addition to its essential role in maintaining homeostasis through the regulation of inflammatory and immune responses, HO-1 is also closely associated with genetic polymorphisms that influence physiological equilibrium (9, 43).

Heme, an iron-rich porphyrin compound, is widely distributed across various cell types and represents the most significant source of iron ions aside from ferritin (44). Its iron ions participate in oxygen binding and electron transfer via redox reactions. However, excessive accumulation of free heme can cause cellular damage, necessitating strict regulation of its intracellular concentration and localization (44, 45). HO-1 plays a crucial role in this regulatory process by catalyzing the oxidative cleavage of heme at the α-methene bridge carbon, yielding biliverdin, carbon monoxide, and free iron ions, thereby precisely modulating the dynamic balance of the intracellular heme pool (9, 43) (**Figure 1**). Biliverdin is converted to bilirubin, a potent antioxidant, while carbon monoxide modulates vasodilation and anti-inflammatory processes (44). In macrophages, both HO-1 and its isoform HO-2 participate in the degradation of heme derived from senescent or damaged red blood cells, thereby playing a crucial role in maintaining systemic heme homeostasis through their enzymatic activities (46). Beyond their central role in maintaining systemic heme homeostasis, HO-1 is also closely linked to ferroptosis. A sequencing-based study has identified *HMOX1* as a crucial ferroptosis-related differentially expressed gene, suggesting that alterations in its expression levels may impact cell survival (8). Notably, HO-1 exerts either cytoprotective or cytotoxic effects depending on the pathological context, thus being characterized as a “beneficial” or “detrimental” molecule (43). An experiment conducted on Apo E knockout mice fed a high-fat diet to induce atherosclerosis, along with *in vitro* studies on macrophage-derived foam cells, has confirmed that HO-1 plays a dual role in ferroptosis: low expression enhances cell viability and reduces lipid peroxidation, whereas high expression can induce ferroptosis in macrophage-derived foam cells (13).

As research progresses, more transcription factors have been identified as regulators of HO-1 expression, with increasing potential for clinical applications. *Nrf2* is a key regulator, promoting HO-1 expression by activating the oxidative stress response (47). In contrast, *BTB and CNC homology 1 (BACH1)* suppresses HO-1 transcription under normal conditions, and stress signals lead to its degradation, allowing *Nrf2* to initiate HO-1 expression (48, 49). Additionally, *activator protein 1 (AP-1)*, *heat shock factor 1 (HSF1)*, *nuclear factor kappa B (NF-κB)*, and *hypoxia-inducible factor 1 alpha (HIF-1α)* contribute to the activation of HO-1 under various stress conditions (49).

In conclusion, the dual role of HO-1 is determined by a complex network of regulatory factors. Currently, its dual effects lack fixed boundaries or a clear definition. Further research is needed to fully elucidate the context-dependent effects of HO-1.

## 4 Regulatory effects of HO-1 on ferroptosis and its implications for atherosclerosis

### 4.1 Iron dysregulation as a driver of HO-1-induced ferroptosis

Iron metabolism plays a critical role in the occurrence and development of iron-related diseases, including ferroptosis. Intracellular iron overload serves as a key factor in ferroptosis (7). Mechanistically, increased iron loading can be achieved through ferritin degradation (ferritinophagy), disruption of iron homeostasis, or the provision of bioavailable iron forms (50). Accumulating evidence indicates that iron metabolism plays an important role in the development of atherosclerosis and HO-1, as a key regulator of iron metabolism, may affect ferroptosis by regulating iron metabolism, and then affect the progress of atherosclerosis.

#### 4.1.1 Modulation of ferritin, ferroportin, and hepcidin: the pathways governed by HO-1

Iron metabolism-related proteins play a critical role in the process of iron metabolism. Typically, iron in the adult body is categorized into two types: functional iron and storage iron (45). Ferritin, a key iron storage protein, sequesters iron in a redox-inactive form and contributes to the regulation of iron homeostasis (51). Simultaneously, ferritin acts as a protective agent by sequestering excess iron, thereby reducing the concentration of free iron in the body, preventing iron overload, and inhibiting ferroptosis (51, 52). Consequently, any alterations in ferritin level can directly influence cellular ferroptosis. Ferritin’s role is complementary to that of ferroportin 1 (FPN1), which serve as a crucial iron transport protein and the only known cellular iron exporter in multicellular organisms, and plays an important role in maintaining systemic iron homeostasis (53). Hepcidin, a peptide hormone synthesized in the liver, binds to FPN1 and induces conformational changes, thereby inhibiting iron export and reducing iron translocation and release within cells (54).

However, overexpression of HO-1 induces the expression of prohepcidin, leading to decreased levels of FPN1 and promoting iron storage in the form of hemosiderin, which reduces cellular iron export and causes excessive iron accumulation within cells (55). At this point, substantial accumulation of free iron exceeds the binding capacity of ferritin, thereby impairing its ability to store iron and disrupting intracellular iron homeostasis. This results in abnormal iron distribution and metabolism (55). This imbalance in iron metabolism not only disrupts the cellular microenvironment but also triggers a cascade of pathological responses, including oxidative stress and inflammation, further exacerbating cellular damage and accelerating disease progression. Notably, in the absence of HO-1, the expression and localization of FPN1 become aberrant (56). Additionally, in breast cancer tissues, HO-1 expression is closely associated with the expression of key iron homeostasis proteins, further highlighting its critical role in regulating iron metabolism (55).

#### 4.1.2 Heme degradation and labile iron release: key roles of HO-1 in iron homeostasis

Heme constitutes a highly bioavailable form of dietary iron, predominantly originating from hemoglobin and myoglobin in meat, poultry, and seafood (45). HO-1 serves as a pivotal enzyme in the heme catabolic pathway, specifically catalyzing the breakdown of heme to release free iron along with other byproducts (42). This process not only represents a fundamental step in heme metabolism but also serves as a critical source of the labile iron pool within cells (57). Increased heme iron intake and elevated body iron stores are associated with an increased risk of cardiovascular disease (58, 59). In the microenvironment of atherosclerotic lesions, chronic inflammation persists as a result of vascular endothelial injury and lipid deposition. Although HO-1 is markedly upregulated in this context, likely serving as an adaptive response to inflammation and oxidative stress, its excessive activation exerts pathological effects during the progression of atherosclerosis. Specifically, HO-1 significantly accelerates heme degradation, leading to aberrant accumulation of free iron in local tissues (13, 59, 60). Free iron that remains unsequestered or unexported can participate in the Fenton reaction, generating hydroxyl radicals and ultimately promoting increased reactive oxygen species (ROS) production and enhanced lipid peroxidation. Collectively, these processes contribute to the onset of ferroptosis (50, 61).

#### 4.1.3 Mitochondrial dysfunction under iron stress: vulnerabilities induced by HO-1

Mitochondria are indispensable for cellular iron utilization and are tightly linked to iron metabolism (62). Beyond their well-recognized role in ATP production, mitochondria orchestrate essential processes such as iron storage, redox regulation, and the biosynthesis of heme and iron–sulfur clusters—key cofactors required for electron transport and metabolic enzyme function (62, 63). When mitochondrial iron homeostasis is disrupted, cellular iron trafficking becomes dysregulated, leading to impaired energy metabolism, excessive ROS formation, and increased vulnerability to ferroptosis (14, 45, 62). These consequences underscore mitochondria as a critical hub in maintaining iron balance and redox stability.

**Figure 3:**
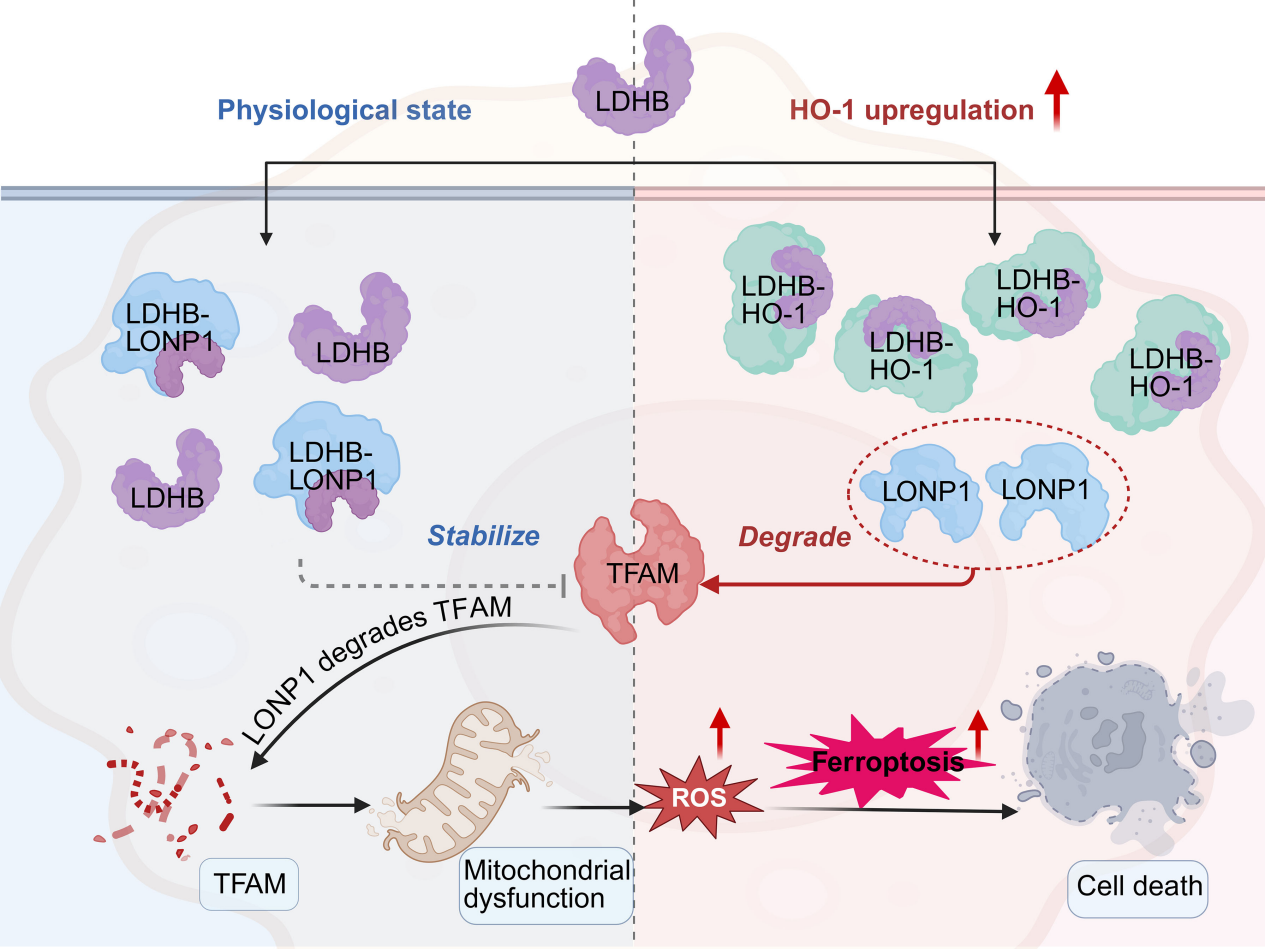
Disruption of mitochondrial function by elevated HO-1 expression. Under physiological conditions, lactate dehydrogenase B (LDHB) interacts with Lon protease 1 (LONP1) to stabilize mitochondrial transcription factor A (TFAM), thereby ensuring mitochondrial function. However, when HO-1 expression is upregulated, it disrupts the LDHB-LONP1 interaction, leading to the degradation of TFAM and mitochondrial dysfunction. This cascade of events results in increased ROS production, triggering ferroptosis and exacerbating macrophage damage within atherosclerotic plaques. (Created in BioRender. Li, D. (2025) https://BioRender.com/00ztb9u).

The mitochondrial transcription factor A (TFAM) is essential for mitochondrial DNA replication and transcription (64). Its stability is tightly regulated by the mitochondrial protease Lon protease 1 (LONP1). Notably, lactate dehydrogenase B (LDHB) has been shown to interact with LONP1, thereby promoting TFAM stabilization and support mitochondrial integrity (64). However, HO-1 and LDHB are highly co-localized within the cell. Under conditions of iron overload, the increased expression of HO-1 competes with LDHB for binding to LONP1, forming a stable complex. This disrupts the interaction between LDHB and LONP1, leading to the accelerated degradation of TFAM (14). This disruption of mitochondrial function further exacerbates oxidative stress and promotes ferroptotic signaling (**Figure 3**). Experimental data show that in this context, mitochondrial ROS and lipid peroxidation levels are elevated, mitochondrial function-related protein levels are decreased, and mitochondrial morphology is significantly impaired (with reduced mitochondrial length and area). However, selective inhibition of HO-1 expression restores these parameters (14). This mechanism is particularly relevant in foam macrophages with low levels of TREM2^low found in advanced atherosclerotic lesions, where genes associated with ferroptosis are notably upregulated (14). These findings suggest a potential role for HO-1 in modulating mitochondrial function and ferroptosis, providing insight into possible therapeutic avenues for late-stage atherosclerosis.

### 4.2 HO-1 and oxidative stress

Oxidative stress refers to a pathological state in which the balance between pro-oxidants and antioxidants is disrupted, leading to the excessive accumulation of ROS in cells or tissues, ultimately causing damage to cellular structure and function (65). Oxidative stress, characterized by excessive ROS, drives ferroptosis, including endothelial injury, lipid infiltration, and inflammatory responses (66, 67). Not only does oxidative stress directly induce cell death, but it also impairs the cellular antioxidant defense system, exacerbating cell damage (68). To maintain redox homeostasis, the body has evolved a complex antioxidant defense system (67). HO-1 exerts bidirectional effects: moderate activation confers antioxidant protection, while excessive HO-1 in conditions of iron overload or chronic inflammation exacerbates ROS accumulation, lipid peroxidation, and ferroptosis (48). Studies have demonstrated that high expression of HO-1 contributes to diabetic atherosclerosis by enhancing oxidative stress and activating ferroptosis, thereby playing a pivotal role in the process of diabetes-related vascular injury (69).

HO-1 exerts a bidirectional regulatory effect on oxidative stress (46, 70). Moderate activation of HO-1 can produce downstream products, such as carbon monoxide and biliverdin, which inhibit the activation of the NOD-like receptor family pyrin domain containing 3 inflammasome, reduce ROS levels, and confer cellular protection (71). However, under conditions of iron metabolism disorder, insufficient antioxidant mechanisms, chronic inflammation, or specific disease models such as atherosclerosis (72), high expression of HO-1 may lead to redox imbalance, further promoting lipid peroxidation and ROS accumulation, thus creating a vicious cycle (14, 46). During atherosclerosis progression, oxidative stress is involved in multiple processes, such as atherosclerotic plaque formation, lipid oxidation, DNA damage, and endothelial dysfunction (67). Moreover, oxidative stress can suppress the activity of the cystine/glutamate antiporter (system Xc-), leading to reduced synthesis and regeneration of GSH, which rapidly depletes its reserves (73). Since the activity of GPX4 is dependent on GSH, a reduction in GSH directly inactivates GPX4, impairing the cell’s ability to detoxify lipid peroxides (74, 75). The collapse of this antioxidant defense system further amplifies the lipid peroxidation cascade, triggering a chain reaction that ultimately disrupts cellular homeostasis (68). As an antioxidant enzyme, HO-1 can effectively reduce ROS levels and inhibit lipid peroxidation, thereby protecting vascular cells from oxidative stress-induced damage (76). However, as atherosclerosis progresses, oxidative stress levels gradually increase. At this stage, high HO-1 expression may disrupt iron ion homeostasis, leading to elevated free iron levels in vascular cells (13). The resulting ferrous ions drive the formation of highly toxic lipid peroxides through lipid peroxidation, exacerbating oxidative stress and initiating the ferroptosis process (13). This process damages the vascular cell membrane and induces cell death, further worsening the pathological changes in atherosclerosis. Notably, oxidative stress can upregulate HO-1 expression by downregulating the transcriptional repressor *BACH1* and promote the translocation of HO-1 from the cytoplasm to the mitochondria, leading to mitochondrial iron overload and the accumulation of lipid ROS (48).

Based on current research, the mechanistic interplay between HO-1 and oxidative stress has been partially elucidated. However, their specific regulatory network and the dynamic relationship under pathological conditions remain unclear, warranting further studies to uncover their molecular mechanisms and biological significance.

### 4.3 HO-1 in plaque stability: protection vs. promotion

Atherosclerosis is a progressive disease characterized by the accumulation of plaques within arterial walls, involving the processes of plaque formation, maturation, and rupture (77). Traditionally, atherosclerotic plaques have been broadly classified into two categories: unstable and stable plaques. Notably, unstable plaques exhibit a pronounced predisposition to intraplaque hemorrhage due to their distinct pathological features, including thin fibrous caps, large necrotic cores, and aberrant neovascularisation (78). Upon plaque rupture, the disruption of the fibrous cap exposes the plaque core to the arterial lumen, triggering a cascade of thrombotic events that may ultimately lead to acute myocardial infarction or cerebral infarction (79). Plaque rupture represents a critical risk factor for acute cardiovascular events and recurrent clinical events following treatment, with its significance far exceeding the influence of plaque size alone (77). Even with anti-inflammatory and lipid-lowering interventions, the rupture of advanced atherosclerotic plaques remains a major risk factor for acute cardiovascular events and post-treatment recurrences (80). Therefore, maintaining plaque stability is a crucial therapeutic strategy to prevent acute cardiovascular events and reduce post-treatment recurrences.

The hallmark features of mature plaques, including iron overload and redox imbalance, serve as indirect evidence for ferroptosis initiation (78). Under the hypoxic microenvironment characteristic of atherosclerotic plaques, the expression of HO-1 in macrophage-derived foam cells is markedly upregulated (81, 82). Studies using mouse models have confirmed that elevated HO-1 not only exacerbates the inflammatory response in macrophage-derived foam cells but also induces ferroptosis, resulting in a significantly increased plaque burden (13). As the primary cell type responsible for inflammatory infiltration within plaques, macrophages exert a direct impact on plaque stability and outcomes through their quantity and phenotypic characteristics (5, 77). A study using ApoE^-^/^-^ Fbn1C1039G+/^-^ mice as a model showed a significant increase in HO-1 expression within carotid plaques (60). This overexpression was associated with the production of matrix metalloproteinases and infiltration of M0 macrophages (8). Matrix metalloproteinases serve as critical enzymes that regulate tissue homeostasis by degrading the extracellular matrix and promoting inflammatory cell infiltration, leading to fibrous cap thinning and plaque instability during atherosclerosis progression (83). M0 macrophages, as resting or unpolarized macrophages, can differentiate into distinct subsets (M1 and M2) in response to alterations in the vascular microenvironment. They play crucial roles in regulating inflammatory responses, foam cell formation, and cell death, ultimately impacting atherosclerotic plaque progression (84). Additionally, systemic oxidative stress may further exacerbate plaque instability and associated risks. Clinical evidence indicates that elevated systemic oxidative stress biomarkers are associated with cardiovascular disease risk (85). Moderate HO-1 activation stabilizes plaques via antioxidative effects, whereas high HO-1 expression promotes macrophage ferroptosis, matrix metalloproteinase activity, and M0 macrophage infiltration, resulting in fibrous cap thinning and plaque instability (71, 86).

### 4.4 Macrophage-induced atypical ferroptosis

Erythrophagocytosis (EP) mediated by macrophages, which induces ferroptosis, represents an important form of non-classical ferroptosis in atherosclerosis (60). The uniqueness of the EP pathway lies in the fact that it is not merely a process of red blood cell (RBC) clearance, but a complex microenvironment of intracellular iron metabolism regulation and redox balance. A key distinction from the classical ferroptosis pathway is that GPX4 levels remain stable, while the expression of HO-1 is significantly elevated, with its regulatory network being more intricate and diverse (60, 87) (**Table 1**).

**Table 1:** Comparison of classic ferroptosis pathway with erythrophagocytosis by macrophages.

Items	Classic ferroptosis pathway	Erythrophagocytosis by macrophages	References
Differences			
Core Mechanism	Reduced cystine uptake, GSH depletion, GPX4 loss	Heme degradation from engulfed red blood cells	(7, 87)
Key Molecule	GPX4	HO-1	(6, 60)
Cell types	Various cells (endothelial, smooth muscle, macrophages)	Phagocytic macrophages	(17, 89)
HO-1 Functions	Heme catabolism, iron homeostasis, mitochondrial function, oxidative stress	Heme degradation, iron release	(14, 17, 60)
Specific Interventions	GPX4 activators, Nrf2 pathway activators	HO-1 inhibitors (zinc protoporphyrin), Hrg1 targeting	(19, 87)
Physiological significance	Local or systemic iron overload, associated with antioxidant defense failure	Local iron overload, associated with erythrocyte clearance	(7, 87)
Similarities			
	Closely related to iron overload, it induces cell death, morphological changes through lipid peroxidation, activates the immune system, triggers inflammation, and is linked to various diseases		(19, 87)

The roles of HO-1 discussed in this table are in the context of promoting ferroptosis.

In atherosclerotic lesions, the rupture of neovessels (or newly formed microvessels) leads to RBC extravasation (88). Macrophages recognize and internalize these RBCs via surface receptors. The macrophage membrane extends to form pseudopodia, enveloping the RBCs and forming phagosomes (87, 89). Subsequently, the phagosomes fuse with lysosomes to form phagolysosomes, where RBCs are degraded by hydrolytic enzymes in the acidic environment of the phagolysosome (87, 90). During this process, the heme responsive gene-1 (Hrg1) is specifically recruited to the phagolysosomal membrane, where it strongly co-localizes with the lysosomal marker Lamp1 (91, 92). Hrg1 facilitates the transport of heme into the cytoplasm, where it undergoes degradation by HO-1 (91). Degradation products such as iron ions, bilirubin, and lipids are recycled by the macrophages, while residual components are secreted into the extracellular space via exocytosis, contributing to local inflammation or tissue repair (89). Notably, *Hrg1* is genetically located upstream of *HMOX1 and Fpn1*, and directly regulates the EP process, further enhancing the complexity of the EP regulatory network (92).

**Figure 4:**
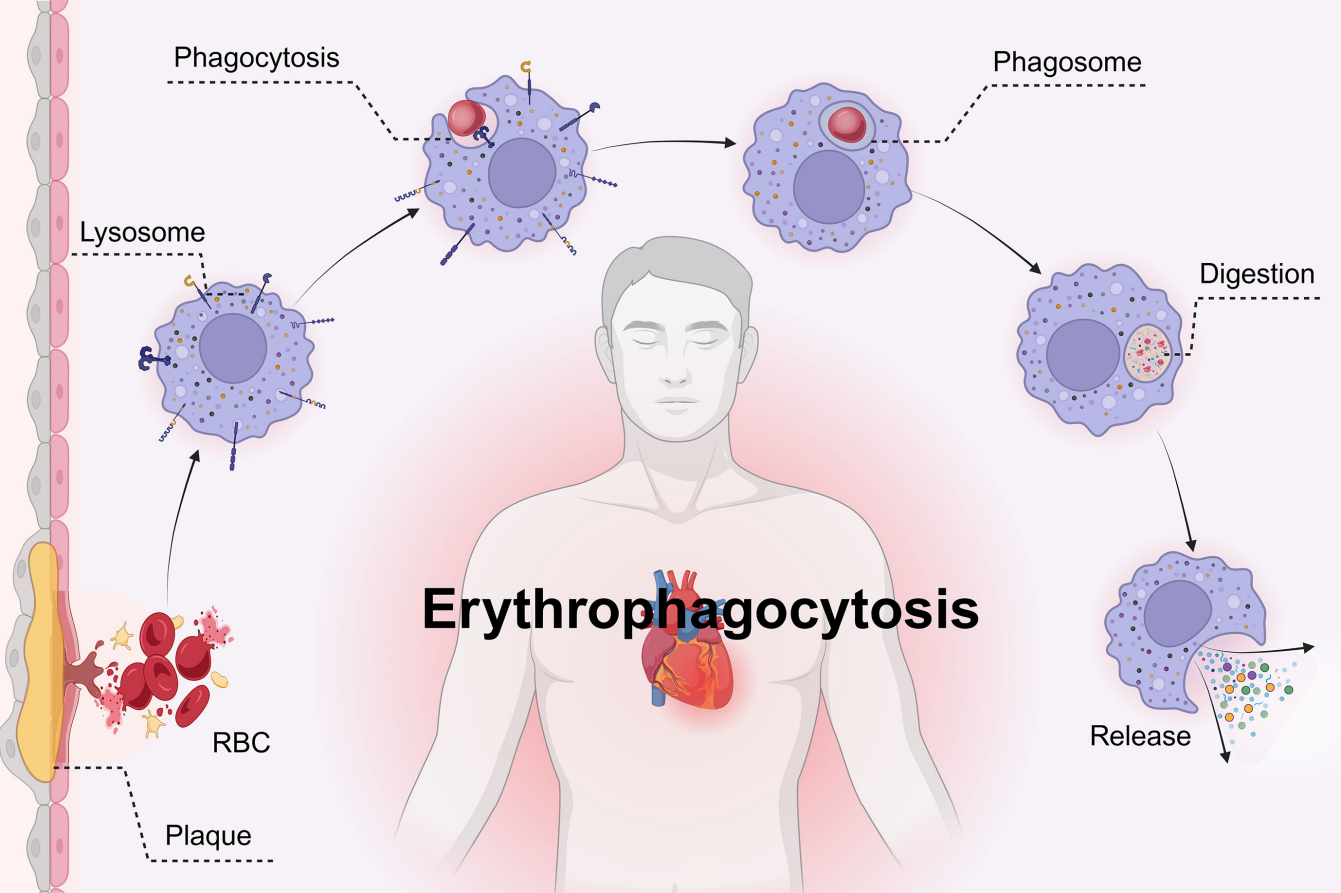
Diagram of the mechanism of macrophage erythrophagocytosis in atherosclerosis. Erythrophagocytosis (EP) refers to the process by which phagocytes, such as macrophages, recognize, engulf, and degrade senescent, damaged, or pathological red blood cells (RBCs) via specific receptors. Within macrophage lysosomes, RBCs are degraded into heme and globin, which are further metabolized into bilirubin and iron. Under normal physiological conditions, EP plays a critical role in maintaining iron recycling and erythropoiesis homeostasis. (Created in BioRender. Li, D. (2025) https://BioRender.com/vsug7vn).

However, in atherosclerosis, intraplaque neovascularisation often exhibits high fragility and permeability due to its immature structure and function (88). These vessels are susceptible to rupture, leading to RBC extravasation and intraplaque hemorrhage (88). In unstable atherosclerotic plaques, RBC fragments are more prominent (16). In such conditions, EP may promote plaque growth by engulfing extravasated RBCs and potentially induce dysfunction in non-professional phagocytes, including vascular smooth muscle cells and endothelial cells, thereby exacerbating atherosclerosis progression (90). Moreover, hemoglobin and its metabolites, released upon RBC lysis, may serve as a significant source of iron within plaques, particularly during the early initiation and advanced stages of atherosclerosis (93). This is supported by findings in bone marrow-derived macrophages, where treatment with RBCs to induce EP resulted in a marked increase in HO-1 expression (approximately 10-fold) (60). The experiments further elucidated hallmark features of ferroptosis, including labile iron accumulation, lipid peroxidation, and cell death, which were effectively inhibited by ferroptosis inhibitors but not by apoptosis or necrosis inhibitors (60). EP contributes to labile iron accumulation, lipid peroxidation, and cell death within plaques, highlighting a novel pathway linking HO-1 to ferroptosis (60, 87). However, the precise role of HO-1 in this process remains unclear and is a subject of ongoing debate. These findings provide additional evidence supporting the hypothesis. Certain studies suggest that during EP, HO-1 not only plays a critical role in RBC detoxification and heme metabolism but also demonstrates antioxidant and anti-inflammatory properties (15). By suppressing inflammation, HO-1 may play a critical role in maintaining erythropoiesis homeostasis (15, 90). Conversely, other studies propose that while excessive HO-1 activation might protect against heme-induced oxidative damage, it may also trigger atypical ferroptosis by generating excessive free iron through heme degradation (60). In the tumor microenvironment, elevated HO-1 expression may even promote malignant tumor progression, though the underlying signaling mechanisms and functional pathways involved remain to be elucidated (15) (**Figure 4**).

The dual role of HO-1 in EP not only depends on free iron levels (including iron intake, storage, and transport) and oxidative stress, but also modulated by additional factors, including phagolysosome processing efficiency, heme transporter levels, and the functional state of macrophages (89, 91, 94). Current research suggests that in macrophages—especially under conditions of impaired phagolysosome function, altered heme transport activity, and oxidative stress—HO-1 may shift from a cytoprotective to a cytotoxic role. This could lead to excessive release of ferrous ions and ROS, thereby triggering ferroptotic cell death. However, additional experimental studies are needed to validate this hypothesis and further investigate the specific mechanisms and pathways involved.

## 5 Conclusions and perspectives

HO-1 plays a dual role in atherosclerosis, particularly in the regulation of ferroptosis, which presents both therapeutic opportunities and challenges (13). On one hand, moderate HO-1 activity exerts protective antioxidative effects, maintaining cellular homeostasis and potentially promoting plaque stability in atherosclerosis. On the other hand, overexpression of HO-1 leads to pathological iron overload, enhanced oxidative stress, and ferroptotic cell death, which can destabilize plaques and increase the risk of adverse cardiovascular events (13, 14). Due to the complexity and vastness of the HO-1 regulatory network, the dual role of HO-1 has yet to be clearly defined or delineated. Both excessive inhibition and activation of HO-1 may carry therapeutic risks, highlighting the difficulty of balancing its dual effects. Effectively suppressing the ferroptotic consequences of HO-1 overexpression, while preserving its beneficial antioxidative function, remains a major challenge in atherosclerosis treatment.

HO-1 can exert both beneficial and harmful effects at different pathological stages, making the regulation of its temporal and spatial expression crucial. For instance, in the late stages of atherosclerosis, inhibition of HO-1 expression effectively mitigates its detrimental effects, such as oxidative stress imbalance, mitochondrial damage, and ferroptosis (12, 14). Additionally, directly targeting ferroptosis or the *HMOX1* gene can significantly alleviate the negative impacts of HO-1. However, targeting HO-1 at any stage may compromise its antioxidant function. Therefore, it is essential to develop targeted therapeutic strategies that selectively modulate HO-1 activity without undermining its protective effects. Current research focuses on counteracting HO-1-mediated ferroptosis through the following strategies: using HO-1 inhibitors (e.g., zinc protoporphyrin-IX (ZnPP)), ferroptosis inhibitors (e.g., ferrostatin-1, iron chelators), modulating key signaling pathways (e.g., targeting the Nrf2/Keap1 axis), and microRNA-mediated inhibition (e.g., miR-24, miR-200c) (69). Additionally, future approaches might integrate advanced drug delivery systems, such as nanoparticles, to locally deliver HO-1 inhibitors to atherosclerotic plaques, thereby improving treatment specificity and efficacy. This approach would allow for precise regulation of HO-1, inhibiting its harmful effects while preserving its antioxidative function. It is important to note that HO-1-mediated ferroptosis has been shown to inhibit the progression of diseases such as glioblastoma, further revealing the potential therapeutic value of HO-1 in other pathological contexts (95). Future research should further investigate the multifaceted functions of HO-1 and assess its therapeutic potential in different pathological backgrounds.

Given that ferroptosis results in cellular rupture and exacerbates inflammation, it remains unclear whether HO-1-induced ferroptosis could establish a positive feedback loop that drives disease progression. While this mechanism is theoretically plausible, the role of HO-1 in different cell types and microenvironments remains controversial, as experimental findings are sometimes inconsistent (9, 12, 15). Furthermore, in the pathological context of atherosclerosis, the interactions between HO-1 and other forms of regulated cell death, such as apoptosis, necroptosis, and necrosis, are primarily confined to the regulatory role of HO-1. Although theoretically, crosstalk between HO-1-driven ferroptosis and these alternative cell death pathways is conceivable, direct experimental evidence remains scarce. Therefore, although HO-1 has demonstrated promising therapeutic potential in cellular and animal models, the clinical application of HO-1 remains uncertain and warrants further experimental investigation.

In conclusion, HO-1 holds significant potential as a therapeutic target for atherosclerosis, but its clinical application faces many challenges. Future research should further clarify the specific roles of HO-1 in different cell types and pathological conditions, and combine clinical studies to assess its therapeutic value in atherosclerosis. Through more comprehensive experimental studies, we aim to provide a theoretical basis for the clinical modulation of HO-1, thus advancing its practical application in atherosclerosis treatment.
